# Preparation of Spray-Dried Soy Isoflavone-Loaded Gelatin Microspheres for Enhancement of Dissolution: Formulation, Characterization and *in Vitro* Evaluation

**DOI:** 10.3390/pharmaceutics6040599

**Published:** 2014-12-08

**Authors:** Gean Pier Panizzon, Fernanda Giacomini Bueno, Tânia Ueda-Nakamura, Celso Vataru Nakamura, Benedito Prado Dias Filho

**Affiliations:** Pharmaceutical Sciences Postgraduate Program, Department of Pharmacy, State University of Maringá, Av. Colombo, 5790, Maringá, 87020-900, PR, Brazil; E-Mails: gean_panizzon@yahoo.com.br (G.P.P.); nanda_farmaco@hotmail.com (F.G.B.); tunakamura@uem.br (T.U.N.); cvnakamura@uem.br (C.V.N.)

**Keywords:** soy isoflavone, microparticle, spray drying, amorphous state, release kinetics

## Abstract

The most bioactive soy isoflavones (SI), daidzein (DAI) and genistein (GEN) have poor water solubility, which reduces their bioavailability and health benefits and limits their use in industry. The goal of this study was to develop and characterize a new gelatin matrix to microencapsulate DAI and GEN from soy extract (SE) by spray drying, in order to obtain solid dispersions to overcome solubility problems and to allow controlled release. The influences of 1:2 (MP2) and 1:3 (MP3) SE/polymer ratios on the solid state, yield, morphology, encapsulation efficiency, particle size distribution, release kinetics and cumulative release were evaluated. Analyses showed integral microparticles and high drug content. MP3 and MP2 yield were 43.6% and 55.9%, respectively, with similar mean size (*p* > 0.05), respectively. X-ray diffraction revealed the amorphous solid state of SE. *In vitro* release tests showed that dissolution was drastically increased. The results indicated that SE microencapsulation might offer a good system to control SI release, as an alternative to improve bioavailability and industrial applications.

## 1. Introduction

Soy isoflavones (SI) are major diphenolic secondary metabolites found in soybeans [*Glycine max* (L.) Merr.] [1]. Soybeans contain basically 12 different isoforms of isoflavones, which are divided into two groups: the aglycone group, including daidzein (DAI) (4,7-dihydroxyisoflavone), genistein (GEN) (4,5,7-trihydroxyisoflavone) and glycitein (4,7-dihydroxy-6-methoxy-isoflavone); and the glycoside group, including daidzin, genistin, glycitin, 6''-*O*-acetyldaidzin, 6''-*O*-acetylgenistin, 6''-*O*-acetylglycitin, 6''-*O*-malonyldaidzin, 6''-*O*-malonylgenistin, and 6''-*O*-malonylglycitin [2]. Almost all isoflavones in soybeans exist in the form of glycosides [3,4]. Interest in these substances is growing, since nutrition research and epidemiological studies in recent decades have suggested that a high dietary intake of soy-containing foods is associated with beneficial biological functions.

SI are known as “phytoestrogens” because they are chemically similar to estrogen and bind to estrogen receptors [5]. Despite the structural diversity found in soybeans, the aglycones DAI and GEN have the most potent and wide-ranging biological activities among SI [6]. These biological activities include the ability to protect against hormone-dependent cancers, increase bone density [7], decrease the risk of heart diseases [8], prevent breast and prostate cancers [9,10], and decrease thrombogenicity and low-density lipoprotein (LDL) levels [11,12]. They may also possess antioxidant [13], serotonin-regulator [14] and antifungal activities [15]. On the other hand, DAI and GEN have rather limited water solubility and dissolve with difficulty in organic solvents. The poor water solubility of these bioactive compounds negatively affects membrane permeation, consequently reducing their bioavailability [6] and also limiting their use for nutraceuticals, functional foods, cosmetics, and pharmaceutical purposes [16,17].

One way to overcome the SI solubility problem is through encapsulation. Various formulations, strategies and production techniques using different polymers, including solvent evaporation using poly(ethylene glycol) [18], complexation with κ-carrageenan [19] or with cyclodextrin [20,21], Fe_3_O_4_-carboxymethylated chitosan nanoparticles, self-nanoemulsified systems [22], superparamagnetic systems and chitosan microspheres [23], have been studied for the encapsulation of SI. However, these procedures showed low encapsulation efficiency and problems with the scale-up process, and would be difficult to apply to large batches. Spray drying is widely used in the pharmaceutical and food industries as a microencapsulation process [24]. The advantages, including easy scale-up, continuous manufacturing and low cost, make spray drying one of the most commonly used solvent evaporation techniques employed as a formulation technology for the enhancement of drug delivery with poorly water-soluble compounds [24]. Thus, microencapsulation with the spray-drying process would be a feasible alternative for encapsulation of SI, avoiding the above-mentioned drawbacks.

Gelatin has been used to encapsulate drugs because it is a natural and biocompatible, inexpensive, and easily available biopolymer. Its biodegradation products are non-toxic and readily excreted. Furthermore, gelatin can be modified by cross-linking, drying, or co-lyophilization, for example, to prepare drug-delivery systems [25].

Microencapsulation of DAI and GEN with gelatin employing spray drying may be able to enhance aqueous solubility and consequently bioavailability, and regulate the release of these phytoestrogens from particles, thus both increasing and modulating the amount released at any given time. This strategy may also play an important role in the health-food industry, by enhancing technological properties and decreasing unpleasant flavors of soy extract (SE).

The aim of this study was to obtain and characterize micronized particles via spray drying, evaluate the effect of processing parameters on the percentage yield and encapsulation efficiency, investigate the effect of microencapsulation with gelatin over DAI and GEN on the solid state of the material, and simulate the gastrointestinal release profile using flow-through cell apparatus.

## 2. Experimental Section

### 2.1. Materials

Analytical standards of aglycones, DAI, GEN and glycitein and the glycosides daidzin, glycitin and genistin were purchased from Tecpar (Curitiba, PR, Brazil) and showed purities of ≥98%. Dried SE (≥40% total aglycone isoflavones) containing colloidal silicon dioxide as excipient were obtained from Galena^®^ (Campinas, SP, Brazil). Pharmaceutical grade gelatin A (Gelita, Maringá, PR, Brazil), distilled water purified by Milli-Q (Millipore Co., Bedford, MA, USA), acetonitrile and acetic acid (HPLC grade; J.T. Baker Chemical Co., Phillipsburg, NJ, USA) and hydrochloric acid (analytical reagent grade) were used. Other reagents and solvents were analytical grade and used as received.

### 2.2. Spray Drying Conditions

Gelatin was initially dissolved in deionized water under magnetic stirring at 65 °C for 45 min to form a gelatin solution (6% or 3% *w*/*v*). SE was suspended in deionized water to obtain dispersions (2% and 3% *w*/*v*). Subsequently, SE dispersions were stirred at 90 °C for 30 min. The final concentration of SE and the polymer ratio were 1:2 (MP2) and 1:3 (MP3) (*w*/*w*). A blank formulation was prepared, excluding SE (MP). Final formulations were spray dried (Mini Spray Dryer, LM MSD 1.0, Labmaq do Brasil, Ribeirão Preto, SP, Brazil) with a 0.7 mm pressurized atomizer nozzle under constant stirring at a feed rate of 12 mL/min. The atomizing airflow rate was 0.4 L/h and the compressed air pressure was maintained at 2.5 Bar. The inlet temperature was controlled at 160 ± 2 °C, and the outlet temperature of 90 ± 7 °C was determined by the inlet temperature and factors, such as air and liquid flow rate. Powders were collected and stored under vacuum at room temperature before characterization.

### 2.3. Characterization

#### 2.3.1. Yield, Morphology and Particle Size Analysis

Yields were gravimetrically determined (type AL204, Mettler Toledo instrument Co. LTD., Shanghai, China) from the final product mass compared to the total amount of spray-dried materials. Morphology and size of the microparticles were examined by scanning electron microscopy (SEM) (Shimadzu SS-550, Kyoto, Japan). SE, blank and loaded microparticles were fixed on double-sided tape attached to an aluminum support, and then coated with gold/palladium under argon atmosphere and examined under a scanning electron microscope. From photomicrographs, the sphericity, roughness of the wall, size of the microparticles and formation of agglomerates were analyzed. Particle size was determined with the aid of an Image-Pro^®^ Plus image analyzer (Silver Spring, MD, USA), by measuring at least one thousand particles present in the photomicrographs. Particle size was characterized by the size below which 10% (*d*(0.1)), 50% (*d*(0.5)) and (*d*(0.9)) of the microparticles were present. MP3, MP2 and MP polydispersity index (PDI) were represented by the distribution span and the mean diameter was taken as the average of *d*(0.1), *d*(0.5) and *d*(0.9) values [26]:

Span = (*d*(0.9) − *d*(0.1))/*d*(0.5) (1)

#### 2.3.2. Encapsulation Efficiency and Drug Content

SE assay, encapsulation efficiency and drug content were evaluated employing the HPLC method and extraction process as described in the quantification of SI in the SE and microparticle formulations. The encapsulation efficiency and drug content of each DAI and GEN in the formulations were determined using the following formulae:

Drug content (mg/g) = mass of DAI and GEN in the microspheres/mass of microspheres
(2)

Encapsulation efficiency (%) = actual drug content/theoretical drug content × 100
(3)

#### 2.3.3. X-ray Diffraction

Patterns were obtained using an X-ray diffractometer (Bruker-AXS, Karlsruhe, Germany). The specifications were 2°/min, 2θ, scanning from 10° to 70°, Cu-Kα X-radiation (λ = 1.5418 Å), 40 mA current and 40 kV voltage.

### 2.4. Quantification of SI in SE and Microparticle Formulations

Isoflavones were extracted from the SE and microparticle formulations, and then quantified. Isoflavones (5 mg) present in the SE and microparticle formulations were placed in tubes containing acetonitrile (10 mL) and shaken (500 rpm × 5 min). Distilled water (6 mL) was added and the mixture shaken again for 60 min. Then, the total volume (25 mL) was completed with distilled water and centrifuged (10,000 rpm × 10 min). The supernatant was filtered through a 0.22 µm membrane, discarding the first 5 mL of the filtrate, and injected in HPLC.

For quantification of SI, a previously validated HPLC analytical method was used. HPLC analyses were carried out on a Shimadzu system composed of a LC-10ADVP quaternary pump, SCL-10AVP controller system, SIL-10ADVP injector, DGU-14 degasser, and software Shimadzu Class VP version 6.12, with a UV–Vis detector (Shimadzu Corporation, Kyoto, Japan). The mobile phase used was: Channel A: 0.1% acetic acid in 18.2 MΩ/cm water (solution A) and Channel B: 0.1% acetic acid in acetonitrile (solution B). The gradient used in Channel B was: 14% (8 min), 14% to 21% (4 min), 21% (3 min), 21% to 29% (5 min), 29% to 40% (3 min), 40% to 50% (2 min), 50% (5 min), 50% to 14% (5 min), 14% (5 min). The injection volume was 20 µL and the UV–Vis detection system was monitored at 254 nm. The flow rate was 1.0 mL/min and the temperature was 30 °C. The separation was performed on a Gemini C18, 250 × 4.6 mm, 5 µm column (Phenomenex Inc., Torrance, CA, USA).

### 2.5. Determination of DAI and GEN in Vitro Release from Formulations

The DAI and GEN *in vitro* release was determined using the flow-through cell method (USP Apparatus 4) (EC 7smart, Sotax Co., Basel, Switzerland). Fifty milligrams of DAI and GEN, calculated from the drug-content results, present in SE and loaded microparticles (MP2 and MP3) were filled on a layer of glass beads in the equipment cells, and then covered with another layer of beads. Glass-fiber membranes with 2 µm pores (Ap25, Millipore, Bedford, MA, USA) were coupled to the cells in order to prevent the passage of undissolved particles in the aliquot to be quantified. Hydrochloric acid 0.1 M (pH 1.2) was used as the dissolution medium, for 60 min. Subsequently there was an automatic exchange of dissolution medium for phosphate buffer pH 7.4 (USP 35) in the remaining 240 min of the test. The dissolution medium flow was 8 mL/min maintained at 37 ± 0.5 °C, and the sampling times were 5, 10, 15, 30, 60, 70, 80, 90, 120, 150, 180, 240 and 300 min. The samples were immediately filtered through 0.22 µm membranes, suitably diluted, and analyzed for isoflavone content using the HPLC method. The release profile of each formulation was characterized by determining the percentage of DAI and GEN dissolved in the medium over time.

### 2.6. Mathematical Modeling

#### 2.6.1. Drug-Release Kinetics

To analyze the release kinetics, data obtained from *in vitro* DAI and GEN release were fitted to mathematical models: zero-order kinetic model (*Q_t_* = *K*_0_·*t*), first-order kinetic model (ln*_Q_* = *K*_1_·*t*), Higuchi model (*Q_t_* = *K*_H_·*t*_1/2_), Korsmeyer–Peppas model (*Q_t_* = *K_k_*·*tn*) and Hixson–Crowell model (*Q*_1/3_ = *K*_HC_·*t*), where *Q_t_* is the amount of drug released at time *t* and *Q* is the amount of drug in the pharmaceutical form at time *t*, *K* is the constant for each model, and *n* is the diffusion or release exponent [27,28].

#### 2.6.2. Release Profile Comparison

The *in vitro* release-profile similarities between formulations MP2 and MP3 were assessed by pair-wise independent-model procedures, such as the difference factor (*f*_1_) and similarity factor (*f*_2_), until 85% release was obtained [27].

### 2.7. Statistical Analysis

The software Statistica^®^ 8.0 (StatSoft, Inc. 1984–2007, Tulsa, OK, USA) was used for statistical analysis. Data are presented as mean ± standard deviation (SD) using a unilateral analysis of variance (one-way ANOVA). Significant differences were determined by Tukey test, with *p* < 0.05 as the significance criterion.

## 3. Results and Discussion

**Figure 1:**
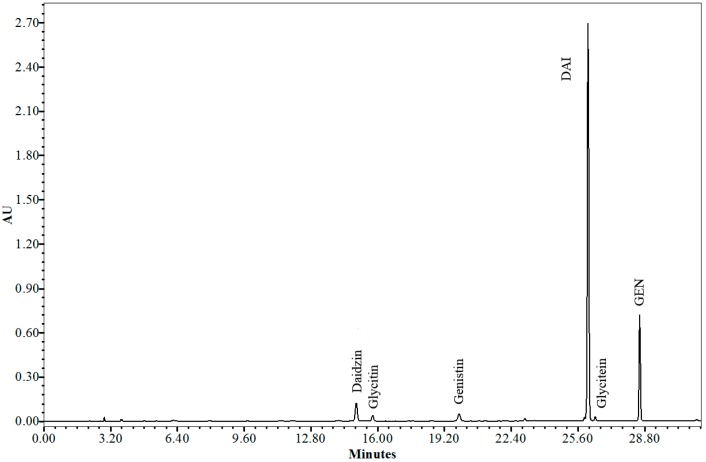
Chromatogram of SE extractive solution. Daidzin (15.5 min), glycitin (15.8 min), genistin (19.9 min), DAI (26.0 min), glycitein (26.5 min) and GEN (28.6 min).

The analytical method for extraction and quantification of SI reported in this study was validated according to the guidelines established by the ICH (International Conference on the Harmonization of Technical Requirements for the Registration of Pharmaceuticals for Human Use) [29] and by Brazilian regulation RE 899/2003 of the National Health Surveillance Agency (ANVISA) [30]. The calibration curve was linear in the concentration range of 1.56–100 µg/mL for both DAI (*y* = 97565*x* + 13311, *r* = 0.999) and GEN (*y* = 185441*x* − 18827, *r* = 0.999). Precision and accuracy were determined from solutions at concentrations of 2, 50 and 100 µg/mL of GEN and DAI, with a coefficient of variation always lower than 5%. Intra- and inter-day precision and accuracy of the method for DAI ranged from 0.3% to 1.5% and 95% to 105%, respectively. For GEN, intra- and inter-day precision and accuracy ranged from 0.20% to 0.24% and from 98% to 102%, respectively. The intra- and inter-day measurements showed that the method is accurate. The limits of quantification and detection for GEN were 0.03 and 0.01 µg/mL, and for DAI were 0.77 and 0.23 µg/mL, respectively. The selectivity was investigated against the formulation components and dissolution medium. No effect was observed on the phytoestrogen retention time, indicating the specificity of the method. The individual amount of SI may vary in the extract, and for this reason the method was also validated for daidzin, glycitin, genistin and glycitein. The glycoside forms, daidzin, glycitin, and genistin, were detected based on the retention times of 15.0, 15.8, and 19.9 min, and the aglycone forms, DAI, glycitein, and GEN were detected based on the retention times of 26.0, 26.5, and 28.6 min, respectively (Figure 1).

The amounts of DAI and GEN in the dried SE were then determined. The SE contained 36.37% ± 0.19% (*w*/*w*) DAI and 4.53% ± 0.08% (*w*/*w*) GEN. The SE analysis showed that the isoflavone content was in accordance with current legislation, fulfilling the specification of ≥40% isoflavone aglycones [27,28,29,30,31]. The content of glycitein in the SE was 0.34% ± 0.01% (*w*/*w*) and the contents of the glycosides daidzin, glycitin and genistin were 2.56% ± 0.01%, 0.72% ± 0.01% and 0.96% ± 0.04% (*w*/*w*), respectively.

### 3.1. Yield, Morphology and Particle Size

The yield and moisture content were (55.9% ± 4.3%)/(4.3% ± 0.5%) (MP2) and (43.6% ± 5.4%)/(6.7% ± 0.7%) (MP3) for 50 g spray-dried solids. The rates of yield of this process may also be associated with the small volume of liquid feed and low content sprayed, and the loss of the smallest and lightest particles [32]. Theoretically, the higher the solid content, the better the yield of the process [33]. However, in the MP3 formulation, the significantly lower yield (*p* < 0.05) may be related to more polymer being deposited on the wall of the equipment. This could be explained due to a high gelatin content, causing an increase in the viscosity, resulting in poor atomization during the process, which would lead to the formation of large and irregular particles, causing accumulation of the polymer on the chamber wall [34,35].

**Figure 2:**
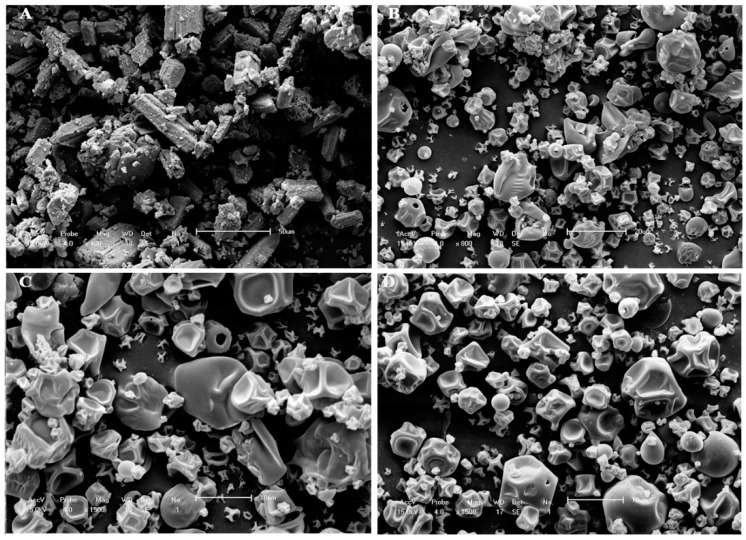
SEM of pure SE (**A**); spray-dried gelatin (**B**); and spray-dried formulations of SE:polymer in the ratio of 1:3 (**C**); and 1:2 (**D**).

SEM of SE clearly indicated a material with irregular shape (Figure 2A). Micrographs of spray-dried gelatin (Figure 2B) showed irregularly shaped microparticles with pinhole and cluster formation. The microparticles from formulations MP3 (Figure 2C) and MP2 (Figure 2D) were spherical, with smooth and uniform surfaces and characteristic concave depressions, and were not coalesced. No structures similar to that in the micrographs of pure SE were observed in the photomicrographs of MP3 and MP2, indicating that SE was completely encapsulated. The addition of SE did not significantly change (*p* > 0.05) the microparticle size distribution. The increase in the SE:polymer ratio from 1:3 to 1:2 (*w*/*w*) did not cause significant visual changes, as shown in scanning electron photomicrographs (Figure 2).

**Figure 3:**
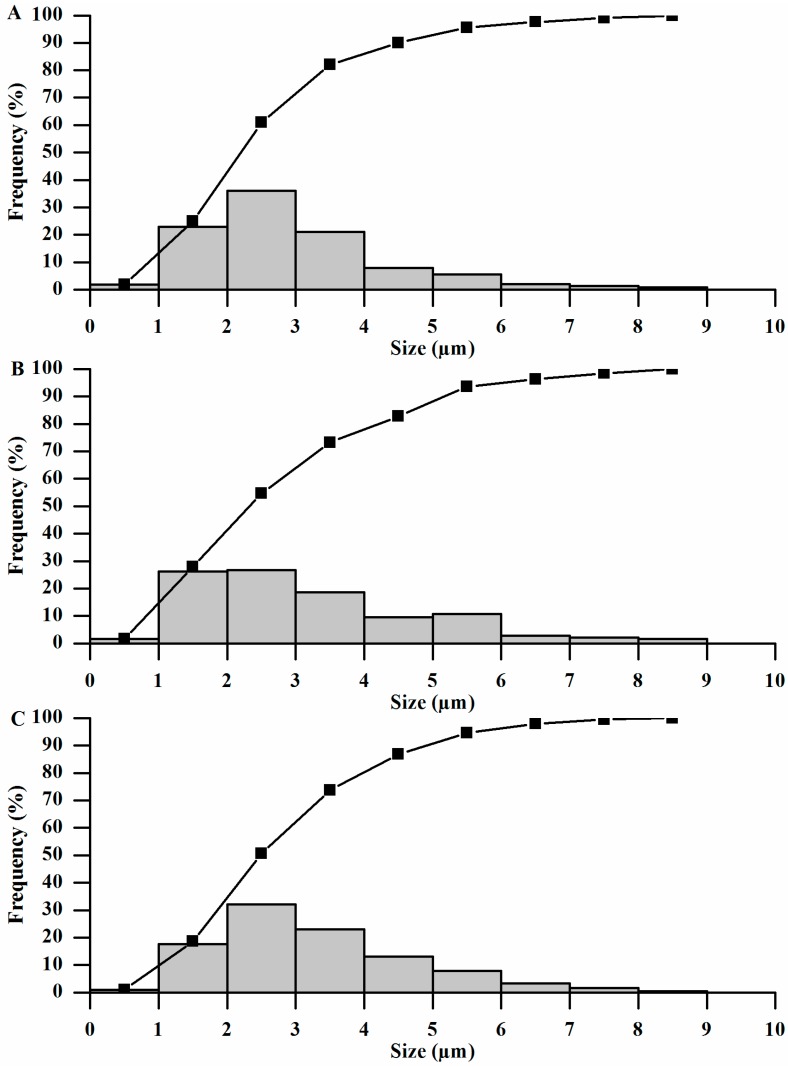
Size distribution of gelatin microparticles: (**A**) MP; (**B**) MP3; and (**C**) MP2. Size frequency distribution (bars) and cumulative size frequency distribution (lines). Frequency: percentage of particle in size interval (1 µm).

Mean particle diameter measured (Figure 3) from the SEM photomicrographs and PDI of the MP, MP2, and MP3, were 3.12, 3.26, 3.31 µm and 1.35, 1.43, and 1.22, respectively. The measurements of microparticles showed similar values (*p* > 0.05) of mean diameter. Moderate distributition was observed for all PDI. These results confirmed that the structures produced were microparticles. Size and morphology and PDI are consistent with the range described in the literature for microparticles prepared by spray drying gelatin-containing propolis [25], ascorbic acid [36], and other plant extracts [32,37].

### 3.2. Encapsulation Efficiency (EE) and Drug Content

The concentration of DAI and GEN found in the microparticles and the experimental results for isoflavones EE in the formulations are shown in Table 1. The very rapid solvent evaporation during spray drying leads to a rapid increase in viscosity, and permits kinetic trapping of isoflavones in the carrier matrix; therefore, satisfactory EE were achieved for MP2 and MP3. A higher EE was achieved for MP3, which could be related to deposition of more polymer on the wall of the apparatus.

**Table 1:** Encapsulation efficiency (%) and drug content (mg/g) from MP2 and MP3. The results are expressed as mean ± SD. Values are means of three independent experiments. SI: soy isoflavon; DAI: daidzein; GEN: genistein; EE: encapsulation efficiency; DC: drug content.

SI	MP3		MP2	
	DC (mg/g)	EE (%)	DC (mg/g)	EE (%)
DAI	90.93 ± 1.67	105.67	121.23 ± 0.27	97.47
GEN	11.33 ± 0.85	95.21	15.11 ± 1.39	90.22
Total	102.26 ± 2.52		136.34 ± 1.66	

### 3.3. Powder X-ray Diffraction (XRD)

**Figure 4:**
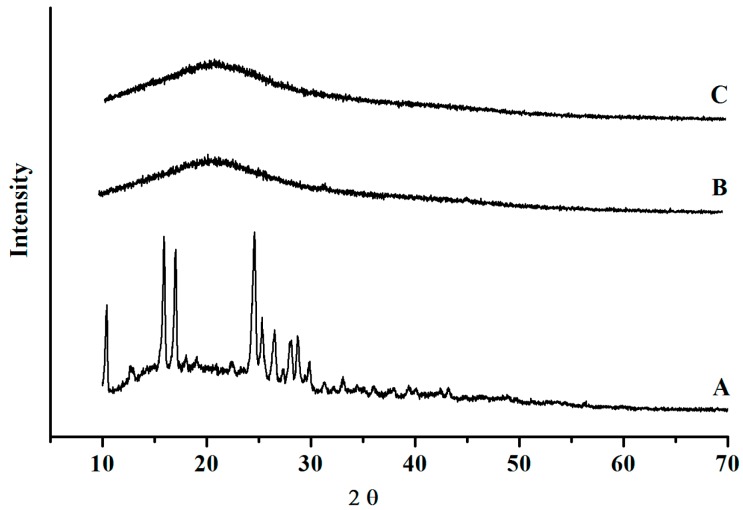
X-ray diffraction patterns of SE (**A**), MP3 (**B**), and MP2 (**C**).

Powder X-ray (XRD) diffraction measurements were used to confirm the crystalline or amorphous nature of the materials. XRD of SE (Figure 4) had the most evident peaks at 2θ = 10.42, 15.92, 17.02, 24.58, 25.30, 26.52, 28.04, 28.74, and 29.88, indicating the presence of crystalline structures. In contrast, under the present formulations and spray-drying conditions, isoflavone-loaded microspheres did not show characteristic peaks, indicating the completely amorphous nature of SE, entrapment, and complexation with the polymer. Substances in the solid state can show crystalline and/or amorphous characteristics. Amorphous solid dispersion is the most energetic solid state of a material [38]. In general, amorphous solids are more soluble than the crystalline forms, because of the free energies involved in the process and dissolution. Solids in the amorphous state have molecules arranged randomly and therefore less or no energy is required to separate them; consequently, they dissolve more rapidly than when in crystalline form [39]. This is an increasingly important approach to formulation, because amorphous solid dispersions have been shown to improve the dissolution rate, solubility, and bioavailability of poorly water-soluble compounds [39,40].

Microencapsulation and complexation employing other techniques and carriers, such as solvent evaporation using polyethylene glycol [18], or complexation with κ-carrageenan [19] and with cyclodextrin [20,21], were also reported to produce some grade of amorphous solid dispersions. However, spray drying is a simple, efficient, robust, fully mature, single-unit operation process capable of evaporating solvents very rapidly, transforming solutions or suspensions into a solid product; and this process has been used industrially for over a century. Recently, due to these characteristics, spray drying has attracted increased interest for the production of viable amorphous solid dispersion products containing poorly water-soluble drugs [24], in order to improve their dissolution [32] and bioavailability.

### 3.4. Determination of in Vitro DAI and GEN Release from SE and Microparticle Formulations

All microparticle formulations released about 25% to 28% of DAI and 41% to 44% of GEN within the first hour. The release profiles of isoflavone-loaded microparticles showed that the formulations were able to drastically increase isoflavone release (*p* < 0.05) compared to pure SE. DAI is more hydrophilic than GEN [41], so the lower cumulative percentage release rate of DAI may be related to the amount of each isoflavone present in the microparticles, since the concentration of DAI was eight times higher than GEN. Another factor may be the mechanism of drug release from the matrix.

**Figure 5:**
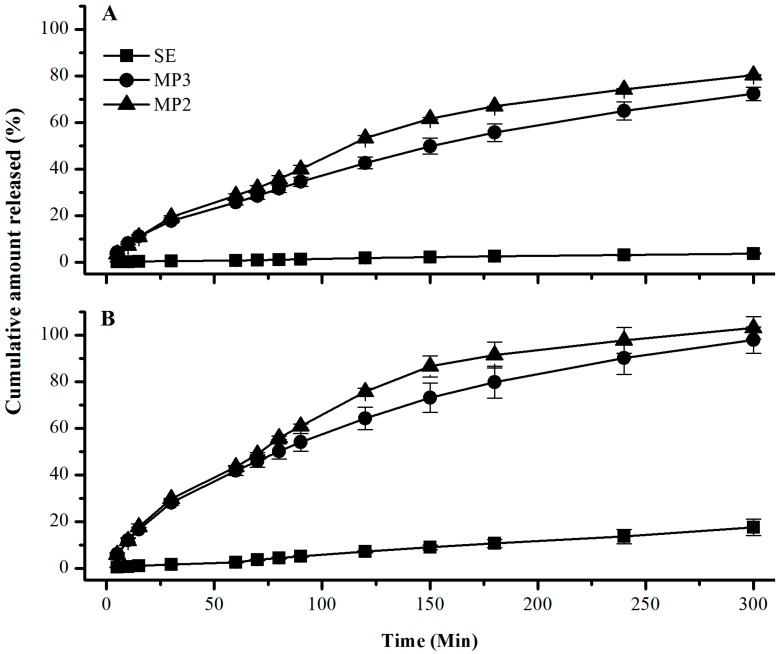
*In vitro* cumulative percentage of DAI (**A**) and GEN (**B**) released (%) from the microsphere formulations (MP2, MP3) and SE in 0.1 M HCl (0–60 min), followed by phosphate buffer pH 7.4 (60–300 min) plotted as a function of time. Values are mean ± SD of 3 independent experiments. DAI: daidzein; GEN: genistein.

As expected, formulation MP2 released significantly larger amounts of SI (*p* < 0.05) than MP3, and almost 100% of DAI and GEN was released after 5 h. Isoflavone dissolution rates for SE were between 0.71% and 2.63% for DAI and GEN, respectively, in the first hour, and remained low until the end of the test (Figure 5). Similar low dissolution rates of commercial SE were also reported by other authors, in an isoflavone-rich extract (90% DAI; 15% GEN) [32] and also for GEN alone [18].

The evaluation of the *in vitro* release profile is an important step because the results are used to guide the development and optimization of formulations or processes, and to predict the behavior of the system *in vivo*. The *in vitro* release stage corresponds to the release of a drug from the dosage form that simulates the drug’s availability for absorption. Oral absorption of any drug occurs in two steps; the drug first dissolves in the gastrointestinal fluid and then permeates across the gastrointestinal membrane. Poorly water-soluble drugs may be absorbed with difficulty, since absorption by the gastrointestinal tract is dependent on dissolution [42]. The release profile of SI was evaluated in a flow-through cell configured in an open mode, where the medium is delivered fresh and the eluate removed. This apparatus was developed to resolve some deficiencies perceived in other compendial techniques [43]. The main advantages over other *in vitro* setups is that the medium can be easily changed, facilitating testing of formulation robustness with respect to variations in the intralumenal environment, and the hydrodynamics are more efficiently simulated [44]. It is also possible to sustain sink conditions. This characteristic is especially important in dissolution testing of poorly soluble drugs, and therefore *in vitro–in vivo* correlations should be easier to establish, because it is possible to obtain the corresponding release in the entire gastrointestinal lumen in a single profile [45].

DAI and GEN are the most responsible for biological activities; because of their physical and chemical characteristics they have low solubility in aqueous media, thus limiting their bioavailability [18,41]. Furthermore, in low doses they can undergo extensive bacterial and hepatic metabolism, which may compromise their systemic availability [46,47]. Microencapsulation of SI may play an important role in regulating the release of isoflavones from pharmaceutical forms, by both increasing and modulating the amount released.

### 3.5. Mathematical Modeling

The data obtained from *in vitro* release tests were analyzed using mathematical models, *i.e.*, zero-order and first-order, and the Higuchi and Korsmeyer–Peppas models to investigate the mechanism of drug release from the microparticles.

### 3.6. Drug Release Kinetics

To predict drug kinetics and release mechanism, data obtained from the *in vitro* release experiments were modeled with different mathematical equations, Zero-order, First-order, Higuchi, Hixson–Crowell and Korsmeyer–Peppas, to determine the mechanism of isoflavone release from microparticles. The values for the release rate constant and regression coefficient (*R*^2^) obtained from the mathematical models are shown in Table 2. The release profiles were not linear, suggesting that the release was not zero order, as confirmed by low *R*^2^ values. Formulations MP3 and MP2 followed first-order kinetics, indicating that the release rate of isoflavones was proportional to the amount remaining in the system. For both formulations, DAI *in vitro* release was best explained by Higuchi’s model based on Fick’s law, where the release is regulated by the diffusion of drugs within the formulation. Initially, hydration of the polymer occurs, followed by absorption of water and desorption of DAI via a swelling-controlled diffusion mechanism [48].

**Table 2:** Release parameters of DAI and GEN from isoflavone-loaded microparticle formulations. SI: soy isoflavon DAI: daidzein; GEN: genistein; *r*^2^: regression coefficient; *K*_0_: Zero-order coefficient; *K*_1_: First-order coefficient; *K*_H_: Higuchi coefficient; *K*_HC_: Hixson–Crowell coefficient; *n*: Korsmeyer–Peppas coefficient.

SI	Test	Zero-Order		First-Order		Higuchi		Hixson–Crowell		Korsmeyer–Peppas	
		*r*^2^	*K*_0_ (mg/min)	*r*^2^	*K*_1_ (min^−1^)	*r*^2^	*K*_H_ (mg/min^½^)	*r*^2^	*K*_HC_ (mg^1/3^/min)	*r*^2^	*n*
DAI	MP2	0.929	0.273	0.991	−0.006	0.986	5.478	0.978	0.007	0.993	0.802
	MP3	0.959	0.239	0.998	−0.004	0.995	4.74	0.992	0.006	0.995	0.698
GEN	MP2	0.874	0.334	0.984	−0.012	0.976	6.87	0.995	0.014	0.989	0.773
	MP3	0.916	0.325	0.981	−0.011	0.995	6.60	0.999	0.011	0.992	0.740

The Hixson-Crowell model was the most suitable for describing the release of GEN. This suggests that the release was not controlled by drug diffusion [27], but mainly by disintegration of the microspheres. The Hixson–Crowell cube root law [49] describes the release from the systems, where it depends on the change in surface area and diameter of the particles with time; it mainly applies to systems that dissolve or erode over time [50] In this case, the GEN release rate is limited by the microparticle erosion rate.

The value of the release exponent “*n*” obtained by applying the Korsmeyer–Pappas equation for the formulations followed anomalous diffusion, *i.e.*, a combination of diffusion and polymer surface erosion-controlled release rate. The contribution of the erosion phenomenon to drug release also increased with the decrease in the amount of polymer. A larger amount of polymer offers more resistance to erosion, and the diffusion process plays a larger role in the release of the drug. The higher percentage of polymer corresponds to a lower porosity of the matrix, which slows the rate of drug release [51,52].

Hydrophilic polymers are the carrier materials that are most often used to prepare solid amorphous dispersions [24]. In general, DAI and GEN release from gelatin microspheres may occur due to a rapid gelation of the matrix as it contacts the water and becomes hydrated. This hydration occurs due to the increase in size of the polymer molecules as a consequence of water uptake, causing a relaxation of the chains. The drug load may also lower the glass transition temperature, indicating plasticization of the gelatin glass, and increase osmotic stress [53]. These processes lead to formation of a zone wherein the polymer passes from a glassy to a gel state. Various phenomena govern transport through this gel layer, for example, the entry of the aqueous medium, where the molecules of the solvent move toward the interior of the polymeric matrix, defining a front of the solvent, with a particular velocity. Simultaneously, intumescence occurs in the opposite direction, and increases with time, removing the drug from the system. Another phenomenon is the unwinding of polymer chains and erosion of the matrix [15].

### 3.7. Comparison of Profile Release

This study investigated the effect of a polymer on the release of soy isoflavones. Because the release profiles appeared visually similar, tests were used to compare the release profiles of these isoflavones between microencapsulated formulations MP2 and MP3. The *in vitro* release profiles of DAI and GEN encapsulated in gelatin microparticles were compared by a pair-wise independent model, by calculating the similarity (*f*_2_) and difference (*f*_1_) factors.

The values of *f*_1_ and *f*_2_ were 13.79 and 58.10 for DAI, and 10.88 and 59.07 for GEN, respectively. Release profiles are judged to be similar to each other when the *f*_2_ and *f*_1_ values range between 50–100 and 0–15, respectively. The results indicate that the release profiles of microencapsulated isoflavones with different amounts of polymers were statistically similar. Comparison of dissolution profiles is useful in cases where it is desired to know the behavior of solid pharmaceutical forms, or before subjecting them to bioavailability tests. This result suggests that the release kinetics of soy isoflavones might not be changed much by the physiological factors in the gastrointestinal tract. Although the *in vitro* release profiles of isoflavones in the microspheres were similar, formulation MP2 provided greater release of DAI and GEN with a smaller amount of microspheres, which is an advantage of this formulation.

## 4. Conclusions

This study demonstrates that gelatin microparticles can be successfully prepared through the spray-drying method to encapsulate DAI and GEN from standardized commercial SE, to obtain an improved powder.

Morphological analysis by scanning electron microscopy revealed micronized particles. The polymer used in the formulations conferred an irregular shape on the microparticles, and this characteristic was not affected by the proportion of polymer used. High drug contents were obtained for both formulations, although MP3 had a lower yield. The results from the *in vitro* release assay clearly indicated that the solubility of DAI and GEN after encapsulation in gelatin was enhanced compared to the native form, and was consistent with XRD measurements indicating a conversion of SI from a crystalline to a completely amorphous form. These results indicate that microencapsulated SE offers a good system to control SI release, and is also an attractive alternative for oral delivery of these poorly water-soluble bioactive molecules.
